# A New Way to Engineer Cell Sheets for Articular Cartilage Regeneration

**DOI:** 10.3390/jfb16120437

**Published:** 2025-11-25

**Authors:** Ta-Lun Tan, Yuan Tseng, Jia-Wei Li, Cheng-Tse Yang, Hsuan-Yu Chen, Her-I Lee, Jun-Jen Liu, Yi-Yuan Yang, How Tseng

**Affiliations:** 1 Ph.D. Program in Medical Biotechnology, College of Medical Science and Technology, Taipei Medical University, Taipei 110, Taiwan; 2 Graduate Institute of Medical Sciences, College of Medicine, Taipei Medical University, Taipei 110, Taiwan; 3 Department of Biomedical Engineering, National Taiwan University, Taipei 106, Taiwan; 4 Department of Biochemistry and Molecular Cell Biology, School of Medicine, College of Medicine, Taipei Medical University, Taipei 110, Taiwan; 5 Department of Orthopedics, National Taiwan University Hospital, Taipei 100, Taiwan; hychen83@ntu.edu.tw; 6 School of Medical Laboratory Science and Biotechnology, College of Medical Science and Technology, Taipei Medical University, Taipei 110, Taiwan; 7 International Ph.D. Program for Cell Therapy and Regeneration Medicine, College of Medicine, Taipei Medical University, Taipei 110, Taiwan; 8 Reproductive Medicine Center, Taipei Medical University Hospital, Taipei 110, Taiwan

**Keywords:** osteoarthritis, cell sheet engineering, non-thermoresponsive, extracellular matrix

## Abstract

Background: Articular cartilage has limited self-repair capacity. While thermoresponsive poly *N*-isopropyl acrylamide (pNIPAAm)-based Cell Sheet Engineering (CSE) is a promising scaffold-free strategy, its inherent material properties pose limitations. This study developed and validated a novel, non-thermoresponsive CSE platform for functional cartilage regeneration. Methods: A culture platform was fabricated by grafting the biocompatible polymer poly gamma-glutamic acid (γ-PGA) and a disulfide-containing amino acid onto porous PET membranes. This design enables intact cell sheet detachment with its native extracellular matrix (ECM) via specific cleavage of the disulfide bonds by a mild reducing agent. Results: The hydrated substrate exhibited a biomimetic stiffness (~16.2 MPa) that closely mimics native cartilage. The platform showed superior biocompatibility and supported the cultivation of multi-layered rabbit chondrocyte sheets rich in Collagen II and Glycosaminoglycans. Critically, in a rabbit full-thickness defect model, transplanted autologous cell sheets successfully regenerated integrated, hyaline-like cartilage at 12 weeks, as confirmed by MRI, CT, and histological analyses. Conclusions: This novel CSE platform, featuring highly biomimetic stiffness and a gentle, chemically specific detachment mechanism, represents a highly promising clinical strategy for repairing articular cartilage defects.

## 1. Introduction

Osteoarthritis (OA) is one of the most prevalent joint diseases in the global aging population. Once considered simple “wear and tear,” OA is now recognized as a “whole-joint disease,” involving a complex pathophysiological process that includes cartilage degeneration, synovial inflammation, and subchondral bone remodeling [1]. The core of OA pathology lies in the destruction of articular cartilage. As an avascular tissue, cartilage possesses a minimal capacity for self-repair [2]. This irreversible damage eventually leads to bone-on-bone friction, causing severe pain and severely limiting patient mobility and quality of life. Current clinical treatments face significant challenges. Non-surgical therapies primarily offer symptomatic relief, have limited efficacy in halting disease progression, and may be associated with adverse effects [3]. Total Joint Arthroplasty (TJA), the definitive surgical solution, is highly effective for pain relief. Still, its limited implant lifespan and the potential risk of revision surgery make it a suboptimal long-term solution for younger, more active patients [4]. Given the inability of existing therapies to reverse joint damage, the development of strategies capable of regenerating functional hyaline cartilage has become a significant and urgent clinical need, driving the field of OA treatment toward regenerative medicine and precision medicine [5].

To address the challenge of cartilage repair, bone marrow stimulation techniques, most notably Microfracture, were adopted as a first-line therapy. This technique involves creating small perforations in the subchondral bone plate to induce mesenchymal stem cells (MSCs) from the bone marrow to form a cell-rich clot, thereby initiating repair [6]. However, this mechanism has fundamental limitations. The induced tissue is predominantly fibrocartilage, which is biomechanically inferior, rather than the native functional hyaline cartilage [6]. The clinical efficacy of this “scar-like” repair is characterized by poor durability; systematic reviews confirm that its benefits often deteriorate significantly within a few years post-operation [7].

Furthermore, this invasive procedure may cause permanent alterations to the subchondral bone, potentially compromising subsequent advanced therapies. In pursuit of higher-quality cartilage regeneration, cell-based therapies emerged, such as Autologous Chondrocyte Implantation (ACI) and its third-generation iteration, Matrix-induced ACI (MACI). The MACI technique involves expanding the patient’s own chondrocytes in vitro and pre-seeding them onto a biological scaffold (e.g., a porcine collagen membrane) before implantation [8]. This FDA-approved approach simplifies the surgical procedure and improves cell retention [8]. Despite this significant advancement, ACI/MACI protocols universally face the “Scaffold Dilemma.” While the exogenous scaffold provides a necessary 3D environment, it may also elicit adverse host inflammatory responses [9] and, more critically, act as a physical barrier impeding the seamless integration of the graft with the surrounding host tissue [9,10].

To eliminate reliance on exogenous scaffolds, a revolutionary “scaffold-free” tissue engineering technology known as Cell Sheet Engineering (CSE) has emerged [11]. Pioneered by Professor Teruo Okano, this technology has become a new paradigm in regenerative medicine [12]. Its core principle relies on culture dishes grafted with the thermo-responsive polymer, poly(*N*-isopropylacrylamide) (pNIPAAm) [13]. At 37 °C, the surface is hydrophobic, permitting cell adhesion; when the temperature is lowered below 32 °C, it transitions to a hydrophilic state [13], facilitating the spontaneous, intact detachment of the entire confluent cell monolayer [12]. This gentle, “enzyme-free harvest” preserves vital biological structures [12,14]. It maintains intact cell–cell junctions, allowing the sheet to function as a pre-organized unit [14]. Most critically, it preserves the basal extracellular matrix (ECM) secreted by the cells [15]. This endogenous ECM acts as a potent “biological adhesive,” [15] enabling the cell sheet to rapidly attach to tissue surfaces without the need for sutures or glues [12,14], thereby overcoming the significant cell loss associated with traditional cell suspension injections [14].

The efficacy of CSE is well-substantiated by extensive evidence. In various preclinical animal models (including rats, rabbits, and minipigs), studies have consistently demonstrated that transplanted cell sheets can induce the regeneration of high-quality tissue that closely resembles native hyaline cartilage in structure and biochemical composition. Histological analysis reveals that the neo-tissue is rich in proteoglycans (Safranin-O positive) and type II collagen, while being deficient in type I collagen, a marker for fibrocartilage [16]. Furthermore, results from human clinical trials in Japan and Taiwan are encouraging [17,18,19]. Patients receiving autologous or allogeneic chondrocyte sheet transplants have shown statistically significant and lasting improvements in knee function scores (KOOS and LKS). Post-operative arthroscopy and MRI have also confirmed that the defects were well-covered with smooth neo-tissue, with biopsies further verifying the formation of high-quality hyaline-like cartilage.

During our review of the field, we explored recent advancements in CSE for OA cartilage repair (summarized in Table 1). As a scaffold-free technology, the core advantage of CSE lies in harvesting cell sheets that retain their intact, highly bioactive ECM. While the autologous cultured chondrocyte sheet (Japan Tissue Engineering Co. (Gamagori, Japan), Product Name: JACC) is approved in Japan [20], its high cost, two-stage surgery, and donor site morbidity have driven research toward allogeneic sources, particularly juvenile chondrocytes (JCCs) [21]. JCCs offer high proliferative capacity and low immunogenicity, enabling the creation of standardized, “off-the-shelf” Master Cell Banks for a single-stage, cost-effective procedure [22].

However, significant translational barriers persist. These include substantial “donor-to-donor variability” and “passage-dependent effects” (e.g., P2 > P9) in JCCs, complicating standardized production [22]. Furthermore, Metzler et al. revealed an “allogeneic blind spot,” noting that pivotal in vivo studies almost exclusively used immunodeficient animal models (e.g., nude mice) [24], thereby bypassing the central hurdle of immune rejection [21]. Interestingly, they also found that in vitro pre-differentiation of JCC sheets offered no in vivo advantage over undifferentiated sheets, strongly suggesting a dominant role for the in vivo microenvironment and opening pathways for simpler manufacturing processes [21].

In summary, CSE represents a tissue reconstruction strategy that more closely aligns with principles of developmental biology. It successfully circumvents the “scaffold dilemma” of traditional tissue engineering, providing a reliable pathway for regenerating high-quality hyaline cartilage. Globally, Japan and Taiwan are at the forefront of the clinical application of this technology, facilitated by their more flexible regulatory frameworks (e.g., Japan’s conditional approval system and Taiwan’s “Regulations for Special Medical Techniques”) [25]. In contrast, the US FDA and European EMA have adopted more stringent approval standards, often classifying such products as advanced therapy medicinal products (ATMPs), which require more extensive clinical data [26,27]. Despite these challenges, as allogeneic cell sheet technology matures, it holds the potential to become a paradigm-shifting therapy for OA.

**Figure 1:**
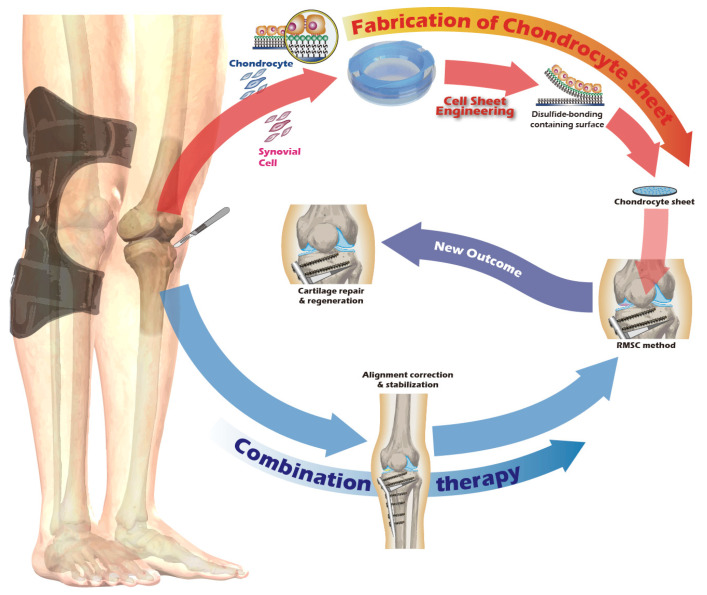
Schematic of the proposed combination therapy for comprehensive knee joint regeneration, integrating cell sheet transplantation with ACI/MACI and an assistive exoskeleton.

Recognizing the remarkable efficacy of CSE, our research group has dedicated years to developing an innovative cell sheet fabrication system that does not require poly(*N*-isopropylacrylamide) (pNIPAAm). This system involves a specialized surface treatment of culture substrates, allowing a confluent cell sheet, complete with its ECM, to be harvested intact through a gentle treatment with a reducing amino acid. Given the urgent clinical need for articular cartilage reconstruction, the present study was designed to apply this non-pNIPAAm-based CSE technology to the field of articular cartilage regeneration. We have designed specialized chondrocyte culture inserts and established a New Zealand white rabbit knee defect model to validate the preliminary therapeutic efficacy of these cell sheets. The ultimate objective of this research is to provide an effective regenerative strategy for patients with knee osteoarthritis and to lay a solid foundation for future human clinical trials. The objective we seek to accomplish is depicted in Figure 1.

## 2. Materials and Methods

### 2.1. Preparation of Cell Culture Inserts

As described in a previous publication by our group [28,29], a surface chemical modification of porous polyethylene terephthalate (PET) membranes was performed. Initially, 0.25 M gamma-polyglutamic acid (γ-PGA) (Vedan, Taiwan) was dissolved in a 0.25 M boric acid buffer (pH 6.0) and mixed overnight. Subsequently, the solution was combined with 1-ethyl-3-(3-dimethylaminopropyl)carbodiimide (EDC) and *N*-hydroxysuccinimide (NHS) powder (ACROS Organics, Geel, Belgium) to create solution A, thereby activating the carboxyl groups of γ-PGA. Concurrently, equimolar cystamine as γ-PGA was dissolved in a 0.25 M boric acid buffer (pH 6.0) to form solution B, which was preactivated for 20 min. Solution A was then added to solution B, and the mixture was allowed to react for 2.5 h at pH 6.0, resulting in solution C.

In the second stage, a 6-well-sized porous PET membrane was pre-activated using a low-pressure plasma apparatus (PDC-002-HP, Harrick Plasma, New York, NY, USA) to introduce reactive functional groups on its inert surface. The pressure was maintained at 400 mTorr for 20 min in an oxygen atmosphere and at 500 mTorr for 45 min in a carbon dioxide atmosphere, with the plasma power set to 45 W. The activated PET membrane was then immersed in a 0.25 M EDC/NHS solution in 0.25 M boric acid buffer at 4 °C for 20 min, followed by the addition of an equal volume of solution C. The reaction was maintained under continuous agitation for 4 h to facilitate covalent bond formation.

Finally, following the surface chemical modification, the disulfide bond-containing γ-PGA-modified porous PET membrane was rinsed gently with deionized water to remove unreacted residues and was subsequently air-dried overnight. The membrane was then affixed to the insert via ultrasonic welding. The assembled culture inserts were sterilized with ethylene oxide gas for subsequent use in cell culture.

### 2.2. Characterization of Surface-Modified PET Membrane

Water contact angle measurements were performed to assess surface hydrophilicity. Prepared films were cut into 20 mm diameter disks and positioned on the instrument stage. An aliquot of 0.2 mL of distilled water was dispensed onto the specimen, and the contact angle was measured using a surface tension meter equipped with CCD dynamic image capture analysis (SEO Phoenix MT, Suwon-si, Gyeonggi-do, Republic of Korea).

The atomic composition and chemical states of the functionalized membrane surface were quantified using Electron Spectroscopy for Chemical Analysis (ESCA/XPS, ULVAC-PHI QuantERA II, Chanhassen, MN, USA) with a 200 µm spot size. Grafted film samples (5 × 5 mm^2^) were positioned on a carrier, and high-resolution spectra of C1s, O1s, N1s, and S2p were acquired to confirm the presence of the grafted molecules and the disulfide linker.

The nanomechanical properties, specifically the elastic modulus (stiffness), of the surface-modified membranes were measured using Atomic Force Microscopy (AFM, Bruker Dimension Icon, Billerica, MA, USA) in PeakForce QNM mode. Measurements were conducted on both dry and fully hydrated samples to assess the effect of hydration on surface stiffness.

For quantitative in vitro experiments (e.g., AFM stiffness and cell proliferation, data are presented as mean ± standard deviation (SD). We used GraphPad Prism v9 software for statistical analysis. Comparisons between groups were performed using Student’s *t*-test or one-way ANOVA. A *p*-value < 0.05 was considered statistically significant. Prior to performing parametric tests, we assessed the data for normality using the Shapiro–Wilk test.

### 2.3. Juvenile Rabbit Articular Chondrocyte Isolation and Cultivation

**Figure 2:**
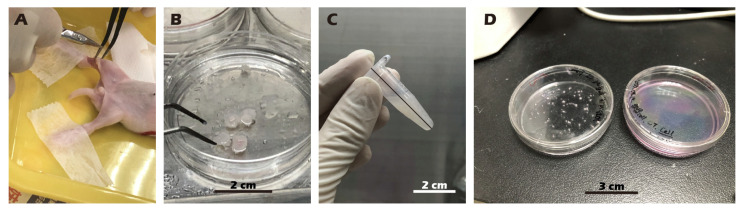
Procedure for harvesting (**A**,**B**), isolating (**C**), and culturing (**D**) chondrocytes from juvenile rabbit knee cartilage. (**A**) Knee cartilage is harvested from an anesthetized and immobilized juvenile rabbit. (**B**) The harvested cartilage tissue. (**C**) The tissue is minced, followed by enzymatic digestion and centrifugation. (**D**) Isolated cells are seeded onto a culture dish.

The experimental unit was a single New Zealand White (NZW) rabbit. NZW rabbit were purchased from the National Center for Biomodels, Taiwan, and housed in a barrier facility. For the in vivo animal study, a total of 6 New Zealand White rabbits were used for the surgical model. In this autologous transplant model, the sample size (*n*) for both the ‘Destroyed’ (left knee) and ‘Cell Sheet-treated’ (right knee) groups was 4. An additional 2 animals were used as non-surgical ‘Control’ animals. All procedures were executed in accordance with institutional guidelines and received approval from the Institutional Animal Care and Use Committee at Taipei Medical University (No. LAC2023-0039). The sample size was determined based on previous, related rabbit model studies for cartilage repair (e.g., [30,31]) and in consideration of the 3Rs principles of animal ethics (Reduction, Replacement, Refinement). A formal a priori sample size calculation was not performed. Exclusion criteria were established a priori and included any pre-existing knee joint pathology or signs of systemic infection identified during pre-operative examination. No animals, experimental units, or data points were excluded from the analysis due to not meeting criteria or post-operative complications. We did not implement explicit strategies to minimize potential confounders, such as the order of surgery or animal cage location. Knee joints were excised from juvenile rabbits, sectioned into small fragments, and homogenized. The particles were treated with Pronase (30 min, 37 °C) and subsequently with Collagenase type II (16 h, 37 °C). After enzymatic digestion, the isolated cells were resuspended in growth medium, filtered through a 70 µm strainer, and cultured on type II collagen-coated dishes at 37 °C in 5% CO_2_. Upon reaching 90% confluency, chondrocytes were passaged, with the medium being replaced every two days. All procedures were recorded as Figure 2.

### 2.4. Rabbit Articular Chondrocyte Isolation and Cultivation

**Figure 3:**
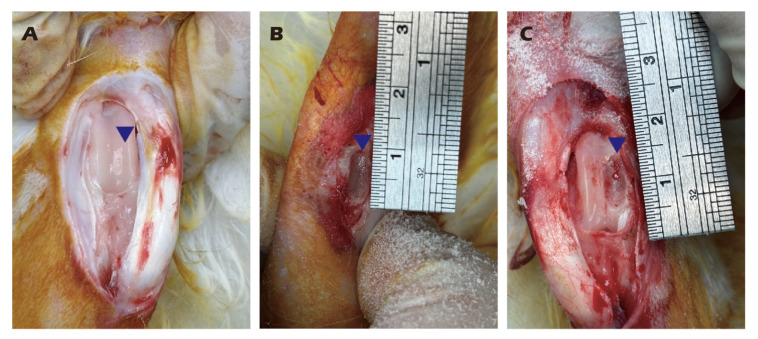
Harvesting of knee cartilage from adult rabbits (**A**,**B**) and creation of a critical-sized articular cartilage defect (**C**). Illustrates the location and dimensions of the defect and the harvested tissue. Blue triangles indicate the surgical site.

Following surgical exposure of adult rabbit knee joints, cartilage was meticulously scraped using a scalpel to obtain homogenized fragments. A full-thickness cartilage defect measuring 1.5 cm in diameter, a critically sized defect for this model, was then created using a dental drill. The harvested cartilage fragments were treated with pronase for 30 min at 37 °C. After removal of pronase by centrifugation (800 rpm, 5 min), the tissue was digested with collagenase type II at 37 °C for 16 h. The collagenase was subsequently removed by centrifugation (800 rpm, 5 min). The isolated cells were resuspended in growth medium (DMEM/F12 with 5% FBS, 30 µg/mL vitamin C, 30 µg/mL insulin, and 1% antibiotic-antimycotic), seeded onto type II collagen-coated dishes, and cultured at 37 °C in a 5% CO_2_ atmosphere. Upon reaching 90% confluency, the primary (P0) chondrocytes were passaged to generate P1 chondrocytes, with medium changes performed every two days. All procedures were recorded as Figure 3.

### 2.5. Preparation and Transplantation of Chondrocyte Sheets

**Figure 4:**
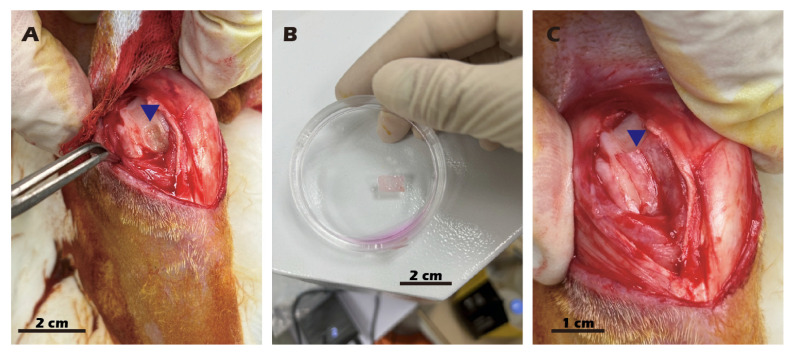
Surgical procedure for autologous chondrocyte sheet transplantation in adult rabbits. (**A**) Creation of the cartilage defect and harvesting of cartilage tissue. (**B**) The harvested cartilage tissue. (**C**) Transplantation of the chondrocyte sheet onto the defect, covered with an occlusive membrane. Blue triangles indicate the surgical site.

**Figure 5:**
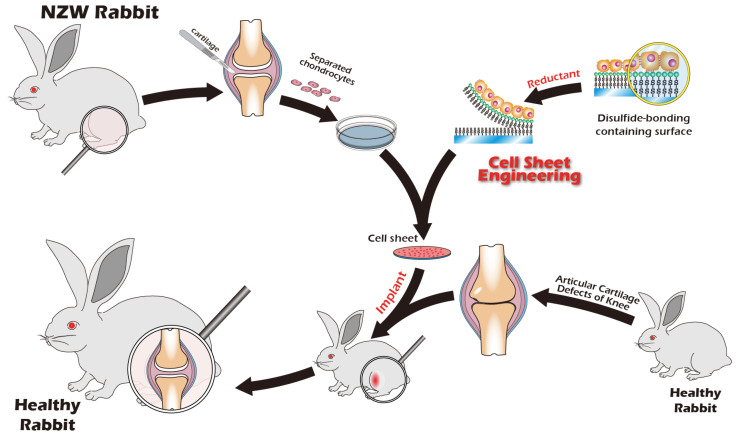
Schematic overview of the autologous chondrocyte transplantation procedure in adult rabbits, from cartilage harvest to cell sheet reimplantation. Cartilage is harvested from the adult rabbit’s knee joint, followed by enzymatic digestion and in vitro cell expansion. A chondrocyte cell sheet is then fabricated using a novel cell sheet engineering technique. This autologous cell sheet is subsequently implanted into the original defect site, facilitating cartilage regeneration over 3–6 months.

P1 chondrocytes were used for the fabrication of cell sheets. The inserts were coated with type II collagen, and 1 × 10^6^ cells were seeded into each insert and cultured for 14 days at 37 °C in a 5% CO_2_ atmosphere. The cell sheets were then detached by adding a 25 mM cystamine solution for 10 min, also at 37 °C in a 5% CO_2_ atmosphere. The first harvested cell sheet was transferred onto a type II collagen-coated tissue culture dish and incubated at 37 °C to allow for attachment. This procedure was repeated twice more to generate triple-layered chondrocyte sheets. A second surgical procedure was performed to expose the previously created defect. The triple-layered cell sheet was trimmed to an appropriate size and transplanted into the target wound. All procedures for preparing the chondrocyte sheet are documented in Figure 4, and the comprehensive diagram outlining the transplantation concept is presented in Figure 5.

### 2.6. Histological and Immunocytochemistry (ICC) Staining

Chondrocytes were fixed with a 4% formaldehyde solution. Standard hematoxylin and eosin (H&E) staining was performed. For ICC, fixed cells on slides were permeabilized with Triton X-100 for 20 min and blocked with 1% bovine serum albumin (BSA) for two hours. Specimens were incubated overnight with primary antibodies against collagen I (1:100, Arigo, Hsinchu, Taiwan), collagen II (1:100, Arigo), and aggrecan (1:100, Thermo Fisher, Waltham, MA, USA). An HRP-conjugated goat anti-mouse IgG (1:100, Arigo) was used as the secondary antibody, followed by hematoxylin counterstaining. For immunofluorescent staining, a FITC-conjugated goat anti-mouse IgG (H + L) (1:100, Arigo) was used as the secondary antibody, with DAPI for nuclear counterstaining.

### 2.7. Chondrocyte Sheet Histological and Immunohistochemical Staining

Chondrocyte sheets were fixed with a 4% formaldehyde solution. For cross-sectional analysis, fixed sheets were processed into paraffin-embedded sections. H&E staining was performed according to conventional methodologies. The same protocols and antibodies described for ICC were used for IHC and immunofluorescence staining of the sheets. For ECM analysis, sheets were fixed in 4% paraformaldehyde. Staining was performed with 1% alcian blue for 30 min, 0.1% safranin O for 5 min (following a 10 min hematoxylin incubation), or 0.04% toluidine blue for 10 min to visualize GAG distribution. Following staining, sections were dehydrated through an ethanol series and cleared with xylene. Staining results were examined using a bright-field microscope.

### 2.8. Magnetic Resonance Imaging (MRI) and Micro-CT Protocol for Ex Vivo Rabbit Knee Specimens

#### 2.8.1. Ethical Approval and Specimen Preparation

All animal procedures were conducted in accordance with institutional guidelines and regulations for the care and use of laboratory animals. The protocol was reviewed and approved by the Institutional Animal Care and Use Committee (IACUC) of Taipei Medical University (Approval No. TMU LAC2023-0039). Following humane euthanasia of New Zealand White rabbits, the hind limbs containing the operated knee joints were immediately disarticulated. To prevent tissue dehydration, each specimen was wrapped in saline-soaked gauze, sealed in a container, and stored at 4 °C. All imaging was completed within 24 h of harvest.

#### 2.8.2. Micro-Computed Tomography (Micro-CT) Imaging

Ex vivo high-resolution imaging was performed using a Skyscan 1176 in vivo micro-CT scanner (Bruker, Kontich, Belgium). Each specimen was immobilized in a non-metallic holder. Scans were acquired with the following parameters: Source Voltage: 80 kVp; Source Current: 280 µA; Resolution: 12.2 µm; Rotation Step: 0.3 degrees; Frame Averaging: 2; Filter: 1 mm aluminum. Raw projection images were reconstructed into 3D datasets using NRecon software v1.6 (Bruker, Billerica, MA, USA) for analysis of subchondral bone morphology, osteophyte formation, and bone microarchitecture.

#### 2.8.3. Magnetic Resonance Imaging (MRI)

Following micro-CT, specimens were imaged using a 7T PharmaScan 70/16 USR MRI system (Bruker, Ettlingen, Germany) with a dedicated rodent knee coil. The multi-sequence protocol included: Sagittal T2-Weighted Fast Spin-Echo (FSE) for evaluating cartilage defects, meniscal integrity, and joint effusion (TR/TE: 2500/33 ms; FOV: 40 × 40 × 20 mm; Matrix: 256 × 256 × 128; Slice Thickness: 0.16 mm; NEX: 4) and Coronal Proton Density-Weighted Fat-Suppressed (PD-FS) FSE for detailed assessment of cartilage morphology (TR/TE: 2500/33 ms). This protocol was selected as T2-weighted imaging is particularly sensitive to changes in tissue water content and the integrity of the collagen fibril network, which are key indicators of cartilage health. DICOM images were analyzed for qualitative and semi-quantitative assessment of cartilage damage using scoring systems such as the International Cartilage Repair Society (ICRS) scale.

## 3. Results

### 3.1. ESCA (XPS) Analysis Confirms Successful Surface Modification and Reversible Detachment Chemistry

**Figure 6:**
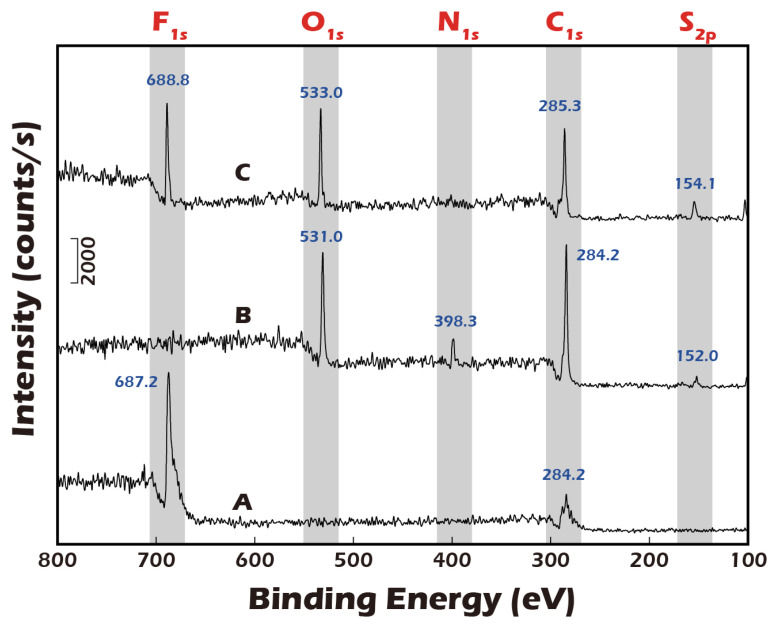
ESCA (XPS) analysis of PVDF membrane surfaces. (**A**) Pristine PVDF membrane. (**B**) PVDF membrane after grafting with disulfide-containing and biopolymer. (**C**) The grafted membrane after treatment with a reducing agent. Successful surface grafting is confirmed by the disappearance of the characteristic F peak (**A**) and the emergence of O, N, and trace S peaks (**B**). The re-emergence of the F peak (**C**) indicates successful cleavage of the disulfide bonds and detachment of the grafted polymer, validating the detachment mechanism.

A foundational requirement of this technology is the precise, multi-step chemical modification of the substrate surface. To validate this process, Electron Spectroscopy for Chemical Analysis (ESCA), a highly surface-sensitive technique, was employed. Figure 6 presents the high-resolution XPS spectra for a polyvinylidene fluoride (PVDF) substrate at the key stages of modification. The pristine PVDF, a fluoropolymer, exhibited a characteristic elemental signature dominated by strong chemical shifts at 687.2 eV, corresponding to the F1s orbital, and at 284.2 eV, corresponding to the C1s orbital of the polymer backbone. Following the plasma treatment and subsequent covalent grafting of the disulfide linker and γ-PGA biopolymer, a dramatic change in the surface chemistry was observed. The prominent F1s peak was almost completely attenuated, indicating that the underlying PVDF substrate was thoroughly covered by a uniform layer of the grafted material. Concurrently, new peaks emerged corresponding to the elemental composition of the grafted layer: a significant O1s peak from the carboxyl and amide groups of γ-PGA, a distinct N1s peak from the amide bonds and amine groups, and a small but crucial S2p peak, which is the definitive signature of the sulfur atoms within the disulfide bond of the linker. This new elemental profile unequivocally confirms the successful grafting of the complete molecular construct. The final step was to validate the detachment mechanism. Upon treatment with a Cysteine solution, which specifically reduces and cleaves disulfide bonds, the surface chemistry was reversed. The XPS analysis showed a strong resurgence of the F1s peak, indicating the re-exposure of the underlying PVDF substrate. Simultaneously, the N1s peak, representative of the γ-PGA layer, disappeared. This dynamic change provides direct and compelling evidence that the disulfide linker was successfully cleaved and the entire grafted polymer-cell complex was lifted off, validating the viability of our chemically induced detachment strategy.

### 3.2. Water Contact Angle Measurements Corroborate Macroscopic Surface Property Changes

**Figure 7:**
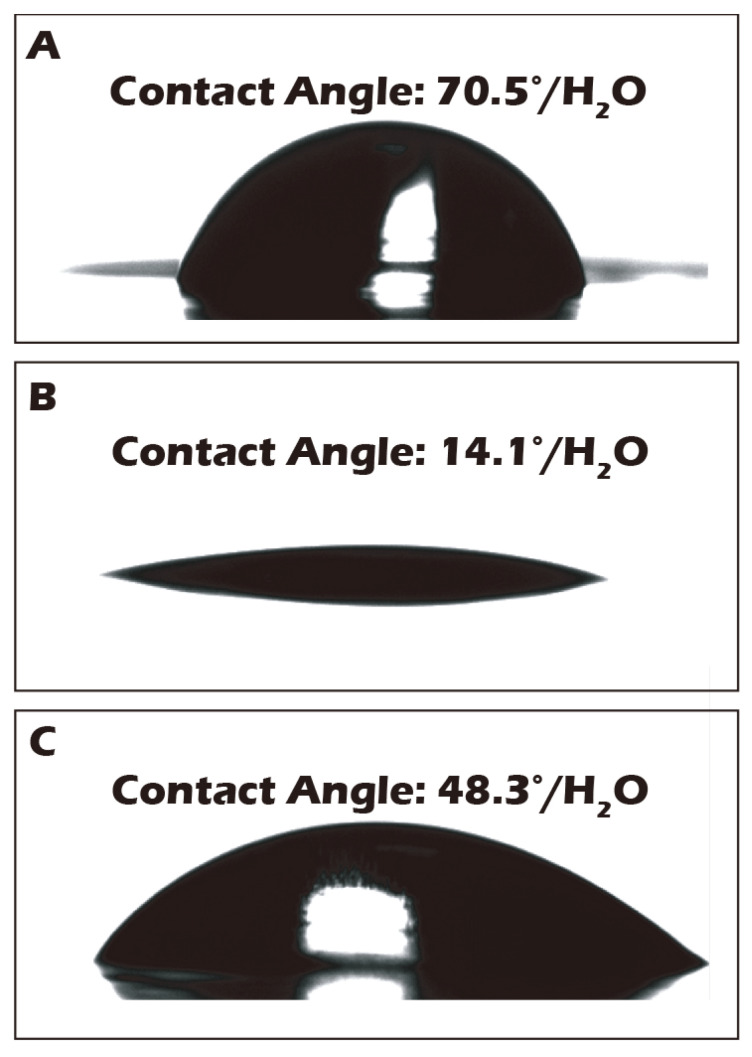
Water contact angle (WCA) measurements of the porous PET membrane surface: (**A**) before grafting, (**B**) after grafting, and (**C**) after reductive treatment. The WCA of the native PET membrane (70.5°) decreased significantly to 14.1° after surface grafting, indicating a substantial increase in hydrophilicity. Following reductive treatment, the WCA increased to 48.3°, further confirming the successful detachment of the hydrophilic polymer layer.

**Figure 8:**
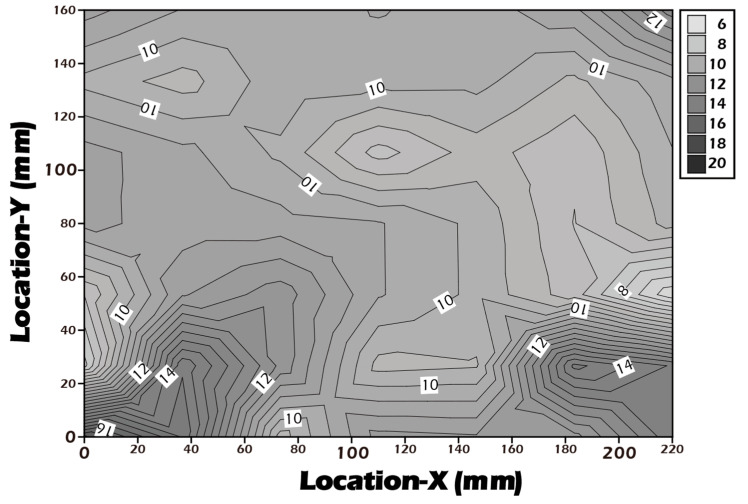
Contour plot of water contact angles measured across a large-area (22 × 16 cm^2^) surface-grafted PET porous membrane, demonstrating grafting uniformity, with WCA values consistently below 16° over the entire surface, indicating a stable and homogeneous surface grafting reaction.

To complement the molecular-level validation from XPS, macroscopic changes in surface properties were assessed using water contact angle measurements, which directly reflect surface wettability and energy. As illustrated in Figure 7, the native polyethylene terephthalate (PET) substrate exhibited a water contact angle of approximately 70.5°, indicating a moderately hydrophobic surface that is generally suboptimal for promoting uniform cell adhesion and spreading. After the chemical grafting of the highly hydrophilic biopolymer γ-PGA, rich in polar carboxyl and amide functional groups, the surface properties were radically altered. The contact angle decreased dramatically to an average of 14.1°, signifying a transition to a highly hydrophilic, high-energy surface. This state is known to be highly conducive to the adsorption of vitronectin and fibronectin from the culture medium, thereby facilitating robust cell attachment and proliferation. Subsequently, to trigger cell sheet detachment, the surface was treated with Cysteine. This cleavage of the disulfide bond and release of the γ-PGA layer resulted in the contact angle increasing to 48.3°. This intermediate value suggests the exposure of a surface less hydrophilic than the γ-PGA layer but more hydrophilic than the original PET, a condition that reduces cell–substrate affinity and aids in the gentle, spontaneous lifting of the intact cell sheet. To ensure the potential for clinical-scale production, where reproducibility is paramount, a multi-point water contact angle analysis was performed on a large-area reacted membrane (Figure 8). The contour plot analysis revealed that the contact angle was uniformly controlled below the target threshold of 15° across the vast majority of the surface area, confirming the high consistency and reliability of our grafting process. This result is a critical quality control metric, indicating that the fabricated culture inserts possess a consistently uniform surface for predictable cell growth.

### 3.3. AFM Reveals Biomimetic Stiffness of the Hydrated Culture Substrate

**Figure 9:**
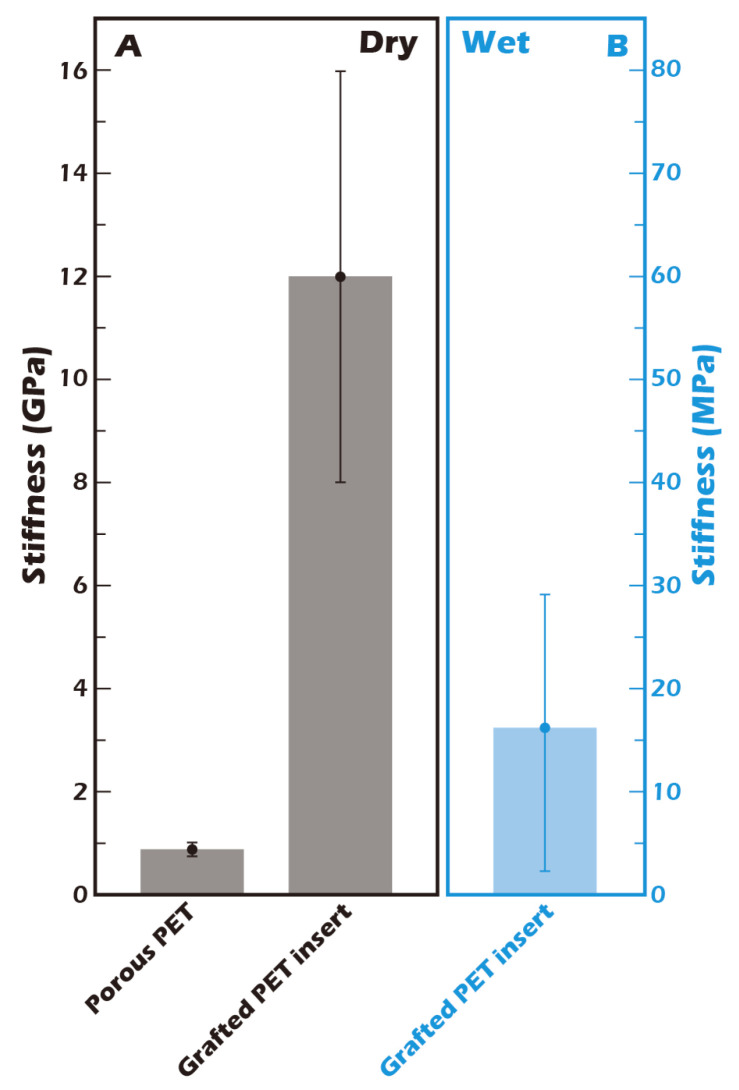
Stiffness measurement of the grafted membrane by Atomic Force Microscopy (AFM) (n = 5) (**A**) in the dry state and (**B**) in a hydrated state. The stiffness of the surface-grafted porous PET membrane increased from 0.8 GPa to 12 GPa in the dry state. However, under hydrated conditions, the stiffness was 16.2 MPa, a value comparable to that of native human articular cartilage [30].

The mechanical properties of the cellular microenvironment are increasingly recognized as critical regulators of cell behavior, a concept known as mechanotransduction. For chondrocytes, substrate stiffness is a key determinant of phenotype maintenance. The stiffness of our culture substrate was therefore quantified by AFM. As shown in Figure 9, the stiffness of the ungrafted porous PET membrane in its dry state was approximately 0.8 GPa. This value increased substantially to approximately 12 GPa after the grafting of the γ-PGA layer, reflecting the formation of a dense, rigid polymer coating. However, the physiologically relevant value is that of the hydrated state. Crucially, when the grafted membrane was fully hydrated, its stiffness decreased precipitously by three orders of magnitude to approximately 16.2 MPa. This dramatic softening is attributed to the plasticizing effect of water on the hydrophilic γ-PGA polymer network. This value of 16.2 MPa closely approximates the compressive modulus of normal human knee cartilage (typically reported in the range of 15–25 MPa) [32]. This finding is of profound biological significance, as it suggests that our hydrated culture surface provides a physiologically relevant mechanical microenvironment for chondrocytes, unlike excessively rigid glass or polystyrene culture dishes. It is hypothesized that this biomimetic stiffness is a key factor in preventing the chondrocyte dedifferentiation into fibroblast-like cells that is often observed on conventional culture substrates.

### 3.4. Grafted Membrane Exhibits Excellent Biocompatibility and Promotes Cell Proliferation

**Figure 10:**
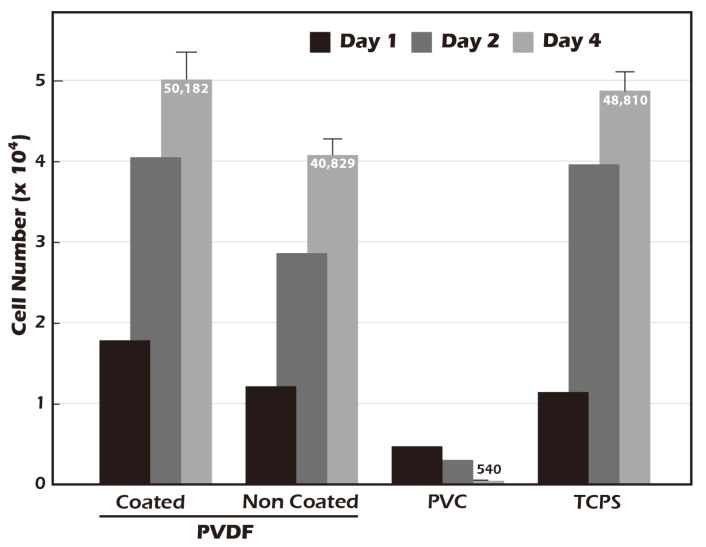
Biocompatibility assessment of the grafted membrane according to ISO 10993-5 guidelines (n = 5). Compared to TCPS (tissue culture polystyrene, control) and PVC (negative control), both unmodified and surface-grafted PVDF membranes exhibited excellent biocompatibility. Notably, the surface-grafted PVDF supported slightly higher cell proliferation than the TCPS control.

A fundamental prerequisite for any material intended for tissue engineering applications is biocompatibility. In accordance with the ISO 10993-5 standard [33], a cytotoxicity test was conducted using NIH3T3 fibroblasts (Figure 10) to assess the biological safety of our modified surface. The cell count for the negative control, polyvinyl chloride (PVC), decreased significantly over the four-day period, confirming its cytotoxic nature. In contrast, both the positive control, standard tissue culture polystyrene (TCPS), and the ungrafted PET membrane supported robust cell proliferation, indicating they are non-toxic. Notably, the surface-grafted PET membrane demonstrated a capacity for cell proliferation that surpassed that of the TCPS control. Cell numbers on our modified surface increased from an initial seeding of 17,911 on day 1 to a final count of 50,182 on day 4, representing a 2.8-fold increase. This was superior to the proliferation observed on TCPS. These results not only confirm that our surface modification process introduces no cytotoxic elements but also suggest that the resulting surface, characterized by its hydrophilicity, biomimetic stiffness, and the presence of γ-PGA (which may act as a nutrient source), actively creates a highly favorable and pro-proliferative environment for cell growth. This enhanced biocompatibility is a significant advantage for producing high-density cell sheets for therapeutic applications.

### 3.5. Cultured Rabbit Chondrocytes Maintain a Stable, Healthy Phenotype

**Figure 11:**
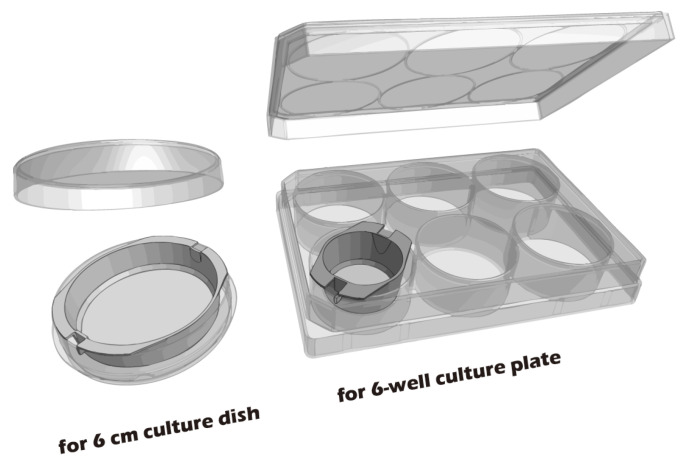
Custom-made culture inserts designed for 6 cm culture dishes and 6-well culture plates. The 6-well plate inserts are intended for small animal models, while the 6 cm dish inserts are designed for large animal or human cell culture.

**Figure 12:**
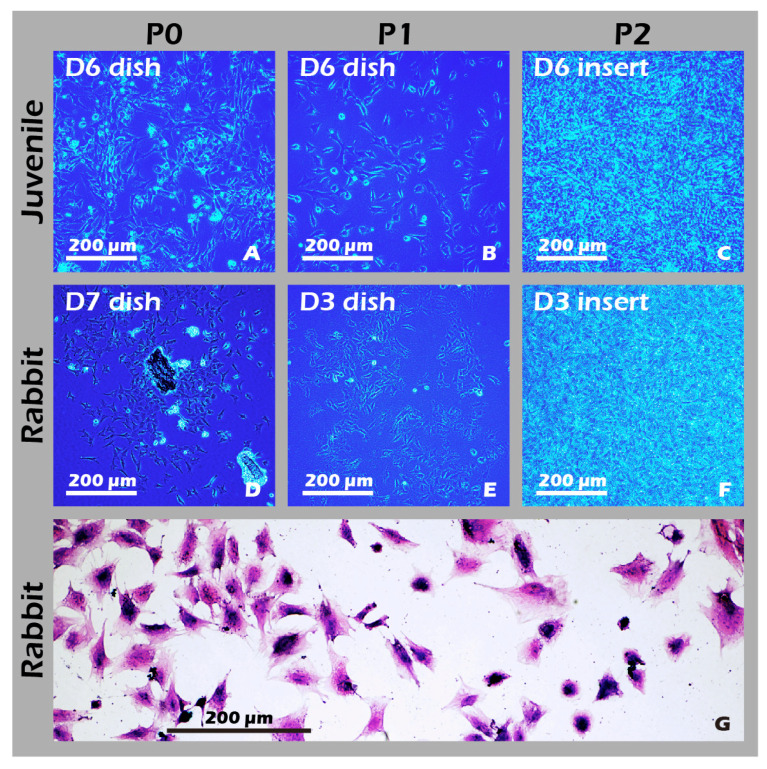
Characterization of cultured chondrocytes. (**A**–**F**) Phase-contrast micrographs (40×) of chondrocytes at (**A**,**D**) passage 0 (P0) and (**B**,**E**) passage 1 (P1) on culture dishes, and (**C**,**F**) passage 2 (P2) on culture inserts. (**G**) H&E staining of fixed P1 chondrocytes (200×). The micrographs confirm that chondrocytes derived from both juvenile and adult rabbits were successfully isolated and are capable of robust proliferation and passaging (subculturing) on both standard culture dishes and custom culture inserts.

**Figure 13:**
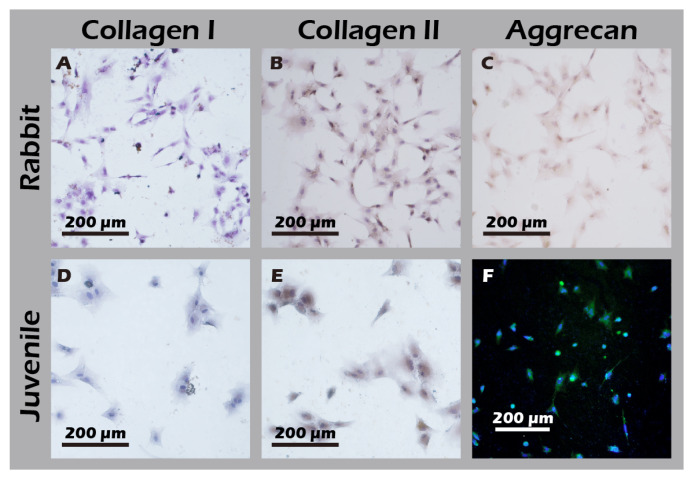
Immunocytochemical analysis of cultured chondrocytes from adult (top row) and juvenile (bottom row) rabbits for (**A**,**D**) Collagen I, (**B**,**E**) Collagen II, and (**C**,**F**) Aggrecan. The first column represents cells from adult rabbits; the second column represents cells from juvenile rabbits. The staining profile (Collagen I-negative, Collagen II-positive, and Aggrecan-positive) indicates that chondrocytes from both sources maintain a phenotype capable of forming hyaline-like cartilage.

To develop culture inserts for future clinical applications, in addition to the 6-well culture plate, a 6 cm culture dish culture insert capable of cultivating large chondrocyte sheets has also been developed. The appearance of these two culture inserts is depicted in Figure 11. To establish a clinically relevant protocol, cartilage was first harvested from juvenile rabbits (1-week-old) as a robust, highly proliferative cell source to validate the system. Subsequently, to better model the clinical scenario of treating age-related or traumatic cartilage damage, cells were also sourced from adult rabbits (>3.5 kg), which typically exhibit a lower proliferative capacity. Chondrocytes from both sources were successfully isolated and expanded on our system. Microscopic evaluation confirmed that cells from both juvenile (Figure 12A–C) and adult (Figure 12D–F) rabbits could adhere and proliferate normally on the grafted PET membrane, displaying the characteristic polygonal, cobblestone-like morphology of healthy, differentiated chondrocytes. H&E staining further confirmed this typical morphology and the formation of a confluent monolayer (Figure 12G). To confirm their phenotype at a molecular level, Immunocytochemistry (ICC) analysis was performed (Figure 13). This analysis demonstrated that chondrocytes from both age groups maintained the correct and desired phenotype: there was strong, uniform expression of Collagen II, the primary structural protein of hyaline cartilage, and Aggrecan, the major proteoglycan responsible for its compressive resistance. Critically, there was minimal to no expression of Collagen I, which is a marker for fibrocartilage and cellular dedifferentiation. These findings are consistent with the definitive molecular signature of healthy, functional articular chondrocytes and confirm that our culture system successfully supports the maintenance of the desired cell type.

### 3.6. Robust Fabrication and Characterization of Multilayered Chondrocyte Sheets with Developed ECM

**Figure 14:**
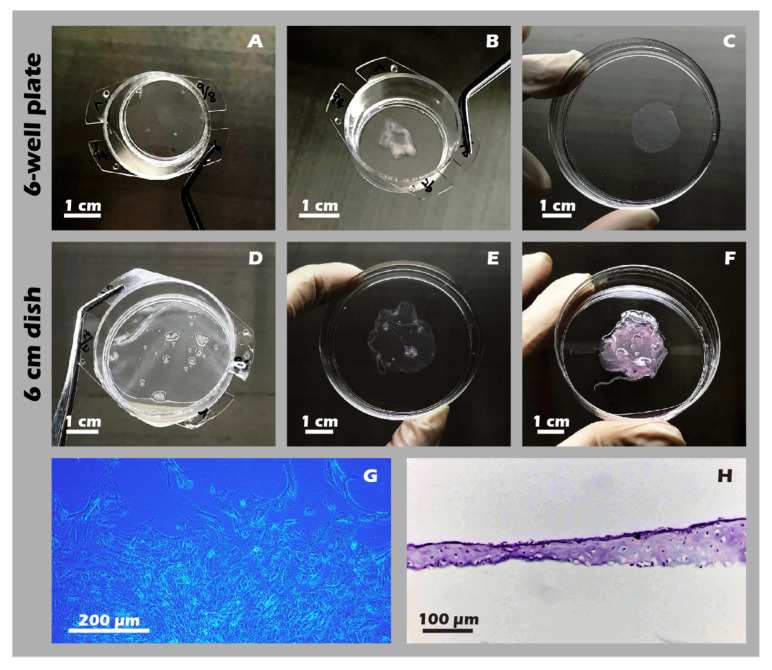
Macroscopic and microscopic appearance of engineered chondrocyte sheets. (**A**) Sheet on a 6-well plate insert before detachment. (**B**) The detached sheet from (**A**). (**C**) The sheet from (**A**) unfolded in a 6 cm dish. (**D**) Sheet on a 6 cm dish insert. (**E**) The detached sheet from (**D**). (**F**) A three-layer stacked construct. (**G**) Micrograph (40×) showing cell outgrowth from a sheet explant. (**H**) H&E staining of a vertical cross-section of a single-layer sheet. These photos show that both single-layer and multi-layer chondrocyte sheets can be readily cultured on and detached from custom inserts, regardless of whether they are designed for 6-well plates or 6 cm dishes (**A**–**F**). Moreover, when the detached cell sheets were re-plated as intact explants onto new culture dishes, they demonstrated successful cell migration and proliferation (**G**), preliminarily indicating the high bioactivity of the fabricated sheets. The resulting cell sheets also exhibited considerable thickness (**H**).

**Figure 15:**
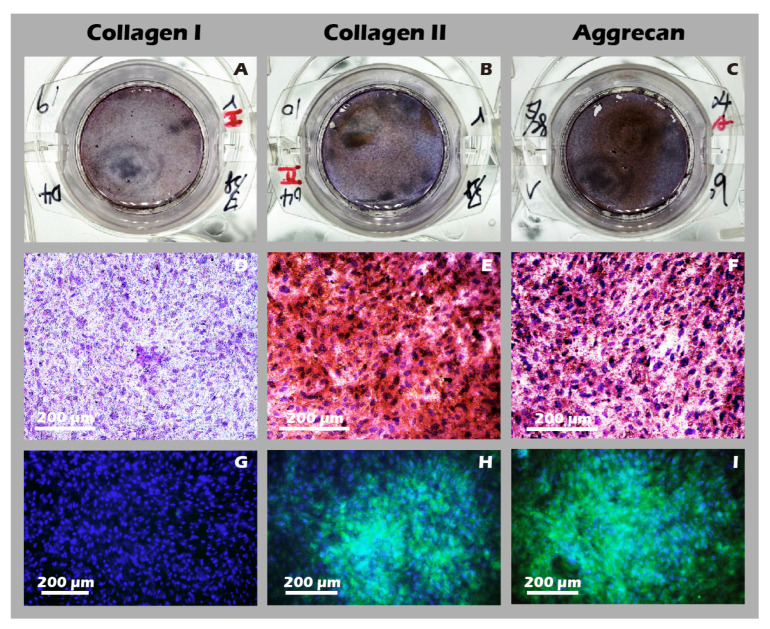
Immunohistochemical (IHC) and immunofluorescence (IF) analysis of chondrocyte sheets. Staining for (**A**,**D**,**G**) Collagen I, (**B**,**E**,**H**) Collagen II, and (**C**,**F**,**I**) Aggrecan. The first column (**A**–**C**) shows macroscopic images, the second column (**D**–**F**) shows IHC micrographs (40×), and the third column (**G**–**I**) shows IF images. The expression profile (Collagen I-negative, Collagen II-positive, Aggrecan-positive) confirms the hyaline-like cartilage characteristics of the cell sheet.

**Figure 16:**
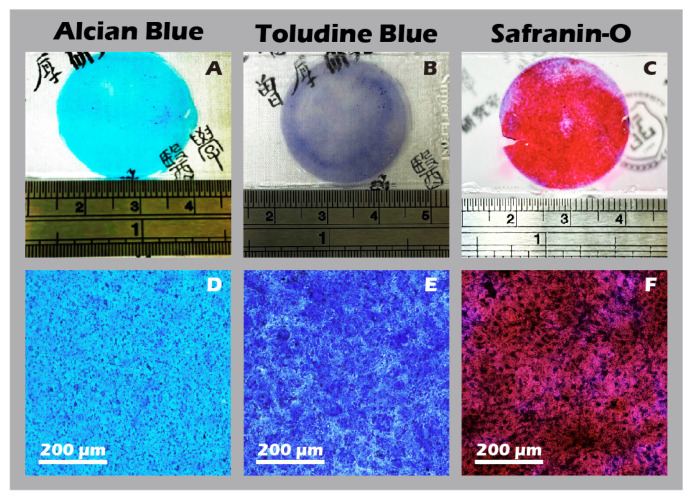
Histochemical staining for extracellular matrix components in chondrocyte sheets. Staining for glycosaminoglycans (GAGs) using (**A**,**D**) Alcian Blue, (**B**,**E**) Toluidine Blue, and (**C**,**F**) Safranin-O. The top row (**A**–**C**) shows macroscopic images, and the bottom row (**D**–**F**) shows corresponding micrographs (40×). Intense positive staining for all three dyes indicates abundant glycosaminoglycan (GAG) production and a well-developed ECM.

Using the developed inserts, viable, cohesive cell sheets were successfully fabricated from adult rabbit chondrocytes. A noticeable, uniform contraction of the cell sheet was observed upon detachment. This phenomenon was attributed to the release of cytoskeletal tension from cell–substrate junctions and the intrinsic contractile properties of the intact, cell-secreted ECM (Figure 14A–F). This contraction is indicative of a well-formed, interconnected tissue construct. The viability and regenerative potential of these detached sheets were confirmed by their ability to give rise to migrating and proliferating cells when transferred to a new culture dish as an explant (Figure 14G), demonstrating their capacity to actively participate in a healing process. Recognizing that many clinical defects require a substantial volume of tissue for repair, we demonstrated that our technique allows for the straightforward stacking of multiple sheets. A construct of three stacked sheets resulted in a tissue of two to three cell layers in thickness, confirming that a sufficient cell mass for transplantation can be achieved (Figure 14H). Comprehensive IHC and IF analyses of these engineered sheets (Figure 15) further confirmed a hyaline cartilage phenotype, with strong, matrix-associated expression of Collagen II and Aggrecan and negligible Collagen I. Furthermore, histochemical staining for GAGs using Alcian blue, Toluidine blue, and Safranin-O (Figure 16) all yielded strong positive results, indicating the presence of a well-developed, proteoglycan-rich ECM distributed throughout the full thickness of the sheet structure. This mature matrix is essential for providing the immediate biomechanical functionality required upon transplantation.

### 3.7. In Vivo Transplantation Leads to Successful Regeneration of Hyaline-like Cartilage

**Figure 17:**
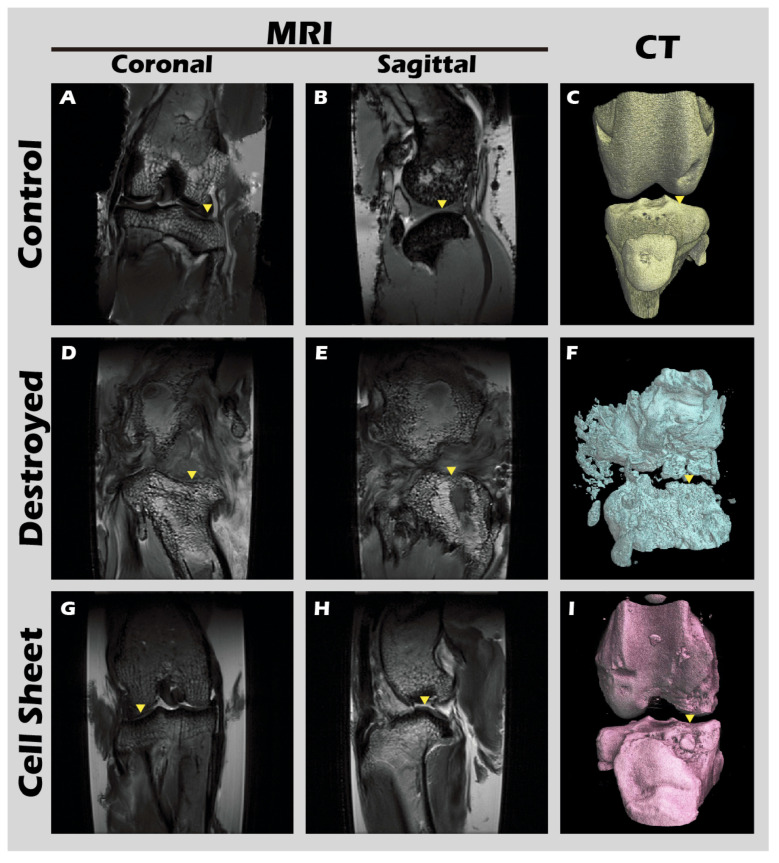
MRI and CT imaging of rabbit knee joints. Representative images from the (**A**–**C**) untreated Control group, (**D**–**F**) defect-only Destroyed group, and (**G**–**I**) Cell Sheet-treated group. First row (**A**,**D**,**G**): coronal MRI scans. Second row (**B**,**E**,**H**): sagittal MRI scans. Third row (**C**,**F**,**I**): 3D-reconstructed CT images. Yellow triangles indicate the defect site. Relative to the Control (**A**–**C**), the Destroyed group (**D**–**F**) exhibits severe degradation of cartilage and subchondral bone at 12 weeks. In contrast, the Cell Sheet treated group (**G**–**I**) shows nearly complete tissue repair.

**Figure 18:**
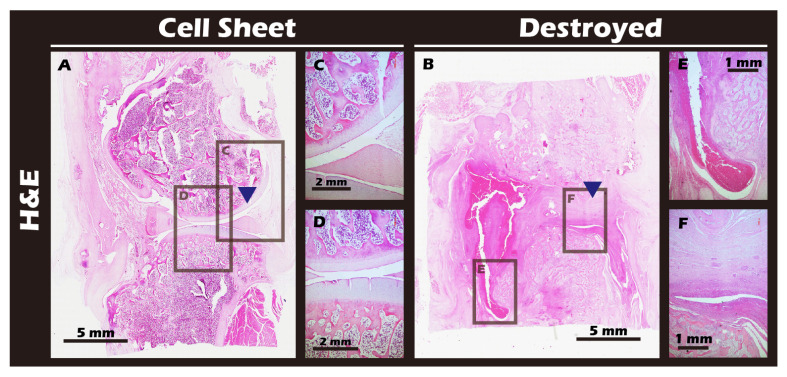
Histological analysis of sagittal sections of knee joints with H&E staining. (**A**) Cell Sheet-treated right knee. (**B**) Defect-only Destroyed left knee. (**C**,**D**) Magnified views of the repaired tissue in (**A**). (**E**,**F**) Magnified views of the defect area in (**B**). Blue triangles indicate the defect site. The Cell Sheet group shows well-defined, regenerated cartilage tissue. The Destroyed group exhibits tissue fusion and loss of distinct articular structures due to cartilage degradation and chronic inflammation.

**Figure 19:**
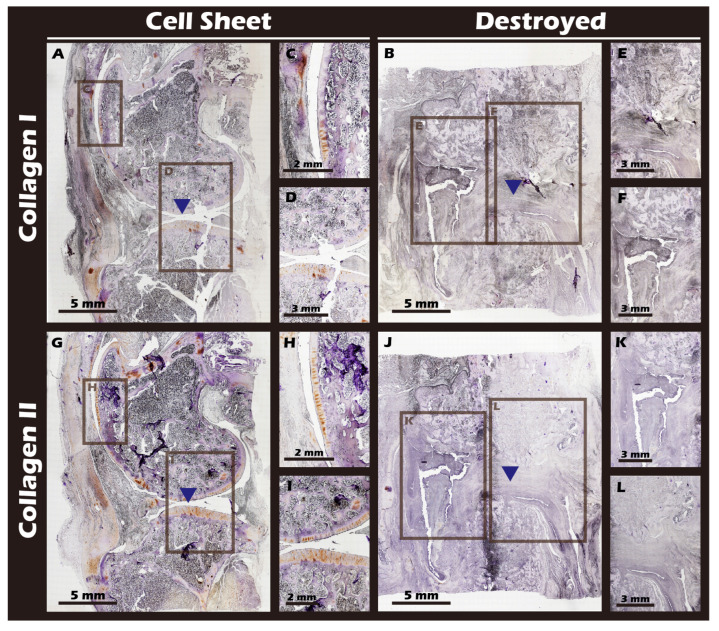
Immunohistochemical staining for Collagen Type I (Col I) and Type II (Col II) in sagittal knee joint sections. (**A**,**G**) Cell Sheet group. (**B**,**J**) Destroyed group. (**C**,**D**) Magnified views of (**A**). (**H**,**I**) Magnified views of (**G**). (**E**,**F**) Magnified views of (**B**). (**K**,**L**) Magnified views of (**J**). The surgical site is indicated by blue triangles. The Destroyed group exhibits strong Col I expression (fibrous tissue) and minimal Col II expression. Conversely, the Cell Sheet group shows negligible Col I staining but significant Col II expression, characteristic of hyaline cartilage.

The ultimate test of our engineered tissue was its ability to regenerate cartilage in vivo. Autologous chondrocyte sheets were transplanted into full-thickness cartilage defects in adult rabbit knees. After a 12-week healing period, comprehensive imaging and histological analyses were performed. High-resolution MRI and Micro-CT scans (Figure 16) provided a clear comparison between the outcomes. The untreated defect in the left knee resulted in extensive pathology, including persistent inflammation, significant tissue degradation, loss of joint space, and the formation of substantial subchondral bone irregularities and osteophytes. In stark contrast, the right knee treated with the chondrocyte sheet exhibited a remarkable degree of healing. The MRI scans showed significant cartilage regeneration, with the repaired tissue appearing nearly restored to the level of the uninjured control, displaying a smooth, continuous articular surface and a normal signal intensity indicative of healthy tissue hydration and composition. H&E staining of the harvested joints (Figure 17) corroborated these imaging findings at the microscopic level, revealing substantial tissue recovery, good surface congruity, and excellent integration with the adjacent native cartilage and subchondral bone in the treated group. Finally, IHC analyses for Collagen II (Figure 18) and Collagen I (Figure 19) were performed to confirm the biochemical nature of the regenerated tissue. The repaired tissue in the treated knee showed strong and widespread expression of Collagen II, characteristic of hyaline cartilage, with a distribution pattern that mirrored the native tissue. Conversely, the untreated defect showed extensive expression of Collagen I, indicative of the formation of non-functional fibrous scar tissue. Collectively, these multi-modal results demonstrate the clear therapeutic efficacy of our autologous chondrocyte sheet in regenerating structurally and biochemically appropriate, hyaline-like cartilage in a challenging large-animal model.

## 4. Discussion

The present study describes the successful development and validation of a novel, patented cell sheet engineering technology. This technology utilizes a disulfide-containing amino acid as a cleavable linker combined with the biocompatible polymer γ-PGA to produce functional, autologous chondrocyte sheets possessing a complete and well-developed extracellular matrix (ECM) [34]. Crucially, it was demonstrated in a New Zealand white rabbit model that these cell sheets can effectively repair damaged knee cartilage, regenerating tissue that is structurally and functionally similar to native hyaline cartilage, thereby offering a highly promising solution for cartilage regenerative medicine.

The prevailing CSE technology relies on the phase transition of the thermoresponsive polymer, pNIPAAm, for cell sheet detachment. However, this mechanism, being a physical phenomenon, is dependent on a specific molecular weight range of pNIPAAm, which consequently constrains the stiffness of the culture surface to a narrow range (approx. 10–500 kPa) [32]. The system described herein employs a distinct chemical mechanism, utilizing the mild reducing agent Cysteine to specifically cleave disulfide bonds for gentle cell sheet release. The core advantage of this design is its high degree of customizability and chemical specificity. By altering the surface biopolymer (in this study, γ-PGA), it is possible to modulate the physicochemical properties of the culture substrate, such as stiffness and charge [35], to more accurately mimic the microenvironment of a specific target tissue [36]. In the past, some studies have indicated that the cells themselves lack strength, different tissues have varying stiffnesses, so the stiffness of the extracellular matrix in different tissues significantly influences cell behavior [37,38,39]. This unique design concept imparts the platform with the potential for broader applications in tissue engineering beyond cartilage [40].

The biomimicry of physical properties is critical for guiding appropriate cell differentiation and proliferation, a process governed by mechanotransduction. The health of cartilage tissue is intrinsically linked to its well-developed ECM, which can constitute up to 80% of the tissue. Our AFM measurements confirmed that the stiffness of our hydrated γ-PGA-grafted membrane is approximately 16.2 MPa, a value remarkably close to that of native human knee cartilage (approx. 20 MPa). In contrast to the hydrophobic state of pNIPAAm systems at a 37 °C culture temperature, our platform provides a more physiologically relevant mechanical environment. It is postulated that this high degree of biomechanical fidelity is a key determinant in the successful cultivation of phenotypically stable hyaline chondrocyte sheets, as appropriate mechanical cues are known to be essential for maintaining chondrocyte phenotype and preventing dedifferentiation via pathways such as YAP/TAZ signaling. Moreover, γ-PGA was selected not only for its excellent hydrophilicity but also based on literature indicating its potential utilization by cells as a nutrient source. This hypothesis was indirectly supported by our cytotoxicity assays, which showed that the grafted PET membrane promoted cell proliferation more effectively than traditional TCPS. The resultant cell sheets stained strongly positive for GAGs, confirming the successful construction of cartilage-like tissue with a developed ECM [41,42,43].

To ensure robustness and potential for future scale-up, a multi-level validation system was established. At the molecular level, high-surface-sensitivity ESCA (XPS) analysis clearly demonstrated the expected changes in elemental composition and chemical states pre- and post-grafting and after Cysteine treatment, confirming the feasibility of the underlying chemical reactions. At the macroscopic level, the significant change in water contact angle (from 70.5° down to 14.1° and back up to 48.3°) provided physical evidence for the mechanism. Importantly, our multi-point analysis of large-area reacted membranes showed uniform contact angles, demonstrating the high consistency and reproducibility of the manufacturing process. This uniformity is a critical prerequisite for Good Manufacturing Practice (GMP) and for providing stable, standardized clinical-grade products in the future [44].

To ensure the potential for clinical translation, the study design closely mirrored clinical scenarios. We first utilized chondrocytes from highly proliferative juvenile rabbits to confirm platform stability, then transitioned to cells from older, adult rabbits, as the target patient population for cartilage reconstruction is typically older or has sustained traumatic injury. Our results showed that chondrocytes from both sources proliferated normally and maintained their correct phenotype. The high transparency of the detached sheets is a hallmark of hyaline cartilage. These sheets not only exhibited excellent viability but also demonstrated the ability to migrate and re-attach in a simulated transplant setting, indicating a strong potential for tissue integration in vivo. Furthermore, our technique allows for the straightforward stacking of multiple sheets, creating a graft 2–3 layers thick, a crucial feature for repairing the deep, often irregular defects found in osteoarthritic joints [31].

The most significant outcome of this research was observed in the autologous transplantation animal study. Through a combination of MRI, CT, and histopathological analyses, it was observed that while the untreated knee joint exhibited severe inflammation and tissue destruction after 16 weeks, the knee treated with autologous chondrocyte sheets for 12 weeks demonstrated almost complete restoration of the continuity, thickness, and smoothness of the cartilage tissue. Strong IHC staining for Collagen II confirmed the regenerated tissue was functional hyaline cartilage, while the minimal expression of Collagen I ruled out the formation of fibrous scar tissue. These results support the conclusion that the engineered autologous chondrocyte sheets can guide true, functional cartilage regeneration in vivo [45].

Despite these encouraging results, the study has certain limitations. Due to the large size of the adult rabbits, longitudinal in vivo imaging with standard small-animal MRI or CT scanners was not feasible. Therefore, our analysis was limited to the endpoint after euthanasia, precluding continuous observation of the repair process. Furthermore, the evaluations in this study were primarily confined to macroscopic and histological analyses, providing an insufficient investigation into the underlying molecular mechanisms of regeneration. We did not present genetic-level expression data (e.g., RT-qPCR or RNA-seq) or protein-level quantitative analyses (e.g., Western blot or ELISA). This limitation, therefore, precludes a more in-depth study of cellular regulatory behaviors and the precise chondrogenic differentiation pathways. Future investigations should not only consider the use of larger animal models, such as pigs or sheep, which more closely approximate the dimensions and mechanical loading of human joints, but also facilitate longitudinal in vivo imaging monitoring. Still, they should also incorporate sampling at critical time points throughout the repair process [30,46]. This would enable more in-depth molecular biology analyses to elucidate the underlying regenerative mechanisms comprehensively

## 5. Conclusions

In summary, the present study successfully developed and validated a novel cell sheet engineering platform based on a disulfide cleavage mechanism. This platform offers multiple advantages, including a highly biomimetic physical stiffness, tunable surface properties, a stable manufacturing process, and a mild, chemically specific detachment condition. Using this platform, autologous hyaline chondrocyte sheets with a well-developed extracellular matrix were successfully cultured from adult rabbit chondrocytes. In a rabbit knee cartilage defect model, we demonstrated that transplantation of these cell sheets could guide the regeneration of structurally and functionally complete hyaline cartilage tissue, achieving excellent repair outcomes. This technology addresses many of the limitations associated with traditional therapies and provides a highly promising clinical solution for treating osteoarthritis and other cartilage injuries, laying a solid foundation for the advancement of regenerative medicine.

## 6. Patents

A relevant patent (US9,546,349 B2) has been issued to H. Tseng and is licensed to Taipei Medical University.

## Figures and Tables

**Table 1:** Recent progress of CSE research on osteoarthritis.

Target Joint/Defect	Cell Type Used	Model	Key Findings and Advancements	Study/Product [Citation]
Knee Joint	Autologous Cultured Cartilage	Clinical (Human)	Approved in Japan for relief of OA symptoms (defect > 2 cm^2^) and for traumatic cartilage defects (defect > 4 cm^2^) in patients refractory to other treatments.	Clinical Application: JACC [20]
Articular Cartilage (Focal Defect)	Juvenile Chondrocyte (JCC) sheets (Allogeneic source)	In vivo (Nude Rat)	In vitro pre-differentiation of JCC sheets did not speed healing. Conventional sheets produced similar hyaline cartilage in vivo, suggesting the environment dominates and a simpler process is possible.	Metzler et al. (2025) [21]
Articular Cartilage (Chondral Defect)	Human Juvenile Chondrocyte (JCC) sheets (P2 vs. P9)	In vivo (Nude Rat)	Confirmed donor-to-donor variability and cell passage dependency affect JCC sheet efficacy, correlating with cartilage regeneration and subchondral bone remodeling.	Matsukura et al. (2023) [22]
Articular Cartilage (Defect Site)	Costal Chondrocytes (CCs) (Scaffold-free TEC)	In vivo (Animal)	Proposes CCs as a cost-effective, direct cell source. Ascorbic acid boosts matrix production, leading to better hyaline-like cartilage regeneration in vivo.	Zheng et al. (2024) [23]
Ectopic Model (Subcutaneous)	Chondrocyte sheet + miR-193b-3p mimics	In vivo (Nude Mice)	In vitro data (not full text) indicate miR-193b-3p is involved in ECM regulation. This in vivo model was used to assess ECM component synthesis post-implantation.	Chen et al. (2019) [24]
Articular Cartilage	Mesenchymal Stem Cells (MSCs)	In vitro	It has been demonstrated that 3D MSC sheets, using a scaffold-free approach, can effectively induce chondrogenic differentiation to produce transplantable hyaline-like cartilage tissue.	Liu et al. (2020) [2]
Articular Cartilage	Autologous Cultured Cartilage	In vivo (NZW rabbit)	An autologous multilayer knee chondrocyte sheet was developed using a scaffolded disulfide bond and gamma-PGA-based culture and detachment system. MRI and CT scans confirmed that this method successfully restored knee cartilage tissue.	Present study

## Data Availability

The original contributions presented in this study are included in the article. Further inquiries can be directed to the corresponding authors.

## Abbreviations

The following abbreviations are used in this manuscript:

CSE	Cell Sheet Engineering
OA	Osteoarthritis
ACI	Autologous Chondrocyte Implantation
MACI	matrix-induced ACI
ECM	extracellular matrix
KOOS	Knee Injury and Osteoarthritis Outcome Score
LKS	Lysholm Knee Score
JCCs	juvenile chondrocytes
ATMPs	advanced therapy medicinal products
pNIPAAm	poly(*N*-isopropylacrylamide)
γ-PGA	gamma-polyglutamic acid
EDC	1-ethyl-3-(3-dimethylaminopropyl)carbodiimide
NHS	*N*-hydroxysuccinimide
PVDF	Poly vinyl difluoride
PET	polyethylene terephthalate
CCD	Charge-coupled Device
ESCA	Electron Spectroscopy for Chemical Analysis
XPS	X-ray photoelectron spectroscopy
H&E	hematoxylin and eosin
ICC	Immunocytochemistry
IHC	Immunohistochemistry
IF	Immuno-fluorescence
IACUC	Institutional Animal Care and Use Committee
Micro-CT	Micro-Computed Tomography
MRI	Magnetic Resonance Imaging
PVC	Poly vinyl chloride
TCPS	Tissue Culture Polystyrene
AFM	Atomic Force Microscope
GMP	Good Manufacturing Practice
